# MicroRNA-182 drives colonization and macroscopic metastasis via targeting its suppressor SNAI1 in breast cancer

**DOI:** 10.18632/oncotarget.13542

**Published:** 2016-11-24

**Authors:** Yun Zhan, Xukun Li, Xiaoshuan Liang, Lin Li, Bangrong Cao, Baona Wang, Jianlin Ma, Fang Ding, Xiang Wang, Da Pang, Zhihua Liu

**Affiliations:** ^1^ State Key Laboratory of Molecular Oncology, National Cancer Center/Cancer Hospital, Chinese Academy of Medical Sciences and Peking Union Medical College, Beijing, China; ^2^ Department of Breast Surgery, Harbin Medical University Cancer Hospital, Harbin, China; ^3^ Department of Breast Surgery, National Cancer Center/Cancer Hospital, Chinese Academy of Medical Sciences and Peking Union Medical College, Beijing, China; ^4^ State Key Laboratory of Bioactive Substances and Function of Natural Medicine, Institute of Materia Medica, Chinese Academy of Medical Sciences and Peking Union Medical College, Beijing, China

**Keywords:** miR-182, SNAI1, colonization, metastasis, breast cancer

## Abstract

Metastasis is a multi-step process. Tumor cells occur epithelial-mesenchymal transition (EMT) to start metastasis, then, they need to undergo a reverse progression of EMT, mesenchymal-epithelial transition (MET), to colonize and form macrometastases at distant organs to complete the whole process of metastasis. Although microRNAs (miRNAs) functions in EMT process are well established, their influence on colonization and macrometastases formation remains unclear. Here, we established an EMT model in MCF-10A cells with SNAI1 overexpression, and characterized some EMT-related microRNAs. We identified that miR-182, which was directly suppressed by SNAI1, could enable an epithelial-like state in breast cancer cells *in vitro*, and enhance colonization and macrometastases *in vivo*. Subsequent studies showed that miR-182 exerted its function through targeting its suppressor SNAI1. Moreover, higher expression level of miR-182 was detected in metastatic lymph nodes, compared with paired primary tumor tissues. In addition, the expression level of miR-182 was negatively correlated with that of SNAI1 in these clinical specimens. Taking together, our findings describe the role of miR-182 in colonization and macrometastases in breast cancer for the first time, and provide a promise for diagnosis or therapy of breast cancer metastasis.

## INTRODUCTION

The process of metastasis consists of a long series of sequential, interrelated steps: dissemination from primary tumors, intravasation, survival and circularization, extravasation, colonization and formation of macroscopic metastases at distant sites [1, 2]. In this process, epithelial-mesenchymal transition (EMT) is always considered as the initial step. Tumor cells undergo EMT to disseminate from primary tumors [3]. A lot of genes and transcription factors, such as SNAI1 [4], Twist [5], ZEB1/2 [6], have been reported to act as key regulators in the EMT process. However, with the in-depth study of tumor metastasis, more and more clinical observations found that macroscopic metastases formed at distant sites within the body typically resemble the primary tumor phenotype, that is, cancer cells reestablish their epithelial identity at the site of metastasis [7]. For example, the epithelial marker E-cadherin, the loss of which represent the occurrence of EMT in some way, its expression was observed to be equal to or stronger in metastatic cancer cells, compared with the corresponding primary tumors [8]. Hence, cancer cells may resemble a mesenchymal-to-epithelial transition (MET), complementary to the initial EMT, to return to an epithelial state [3, 9].

Many microRNAs (miRNAs) have been reported to act as metastasis promoters [10, 11] or suppressors [12, 13]. Some of them have been demonstrated to enhance or repress metastasis by regulating the EMT process, such as miR-200 [14], miR-34 [15] and miR-9 [16]. However, some other reports have observed contrary results correlated with previous findings on the molecular mechanisms underlying EMT. For example, miR-200, a well-known EMT suppressor [14, 17, 18] has been reported to enhance colonization to form distant metastasis in breast cancer [19, 20]. Besides, miR-155 could prevent 4T1 cells from undergoing EMT and reduce dissemination of the tumor cells to the lung, whereas promoting macroscopic tumor formation in the lung via tail vein injection [21]. These findings indicate that EMT-related miRNAs may result in entirely different metastasis process via regulating EMT at multiple steps of metastasis. The roles of EMT-related miRNAs in early stage of metastasis are widely known. Even so, less is known about their influence on colonization and macrometastases formation at distant organs, whereas the reversion of EMT, is considered to be essential to establish macrometastases [22].

Therefore, to identify more EMT-related miRNAs and investigate their functions on colonization and macrometastases formation, we established microRNA and mRNA microarray profiles in SNAI1-induced EMT in MCF-10A cells *in vitro*. We characterized that microRNA-182 (miR-182), which was directly suppressed by SNAI1, could enable an epithelial-like state in breast cancer cells. Meanwhile, miR-182 served as a promoter in colonization and macrometastases formation at distant organs *in vivo* via targeting SNAI1. Moreover, compared with matched primary breast cancer tissues, miR-182 increased in metastatic lymph nodes, while SNAI1 decreased in these lymph nodes. Taken together, we demonstrate that miR-182 acts as a promoter of colonization and macrometastases formation in breast cancer, providing a promise for diagnosis or therapy of breast cancer metastasis.

## RESULTS

### Identification of miR-182 in metastasis process

**Figure 1:**
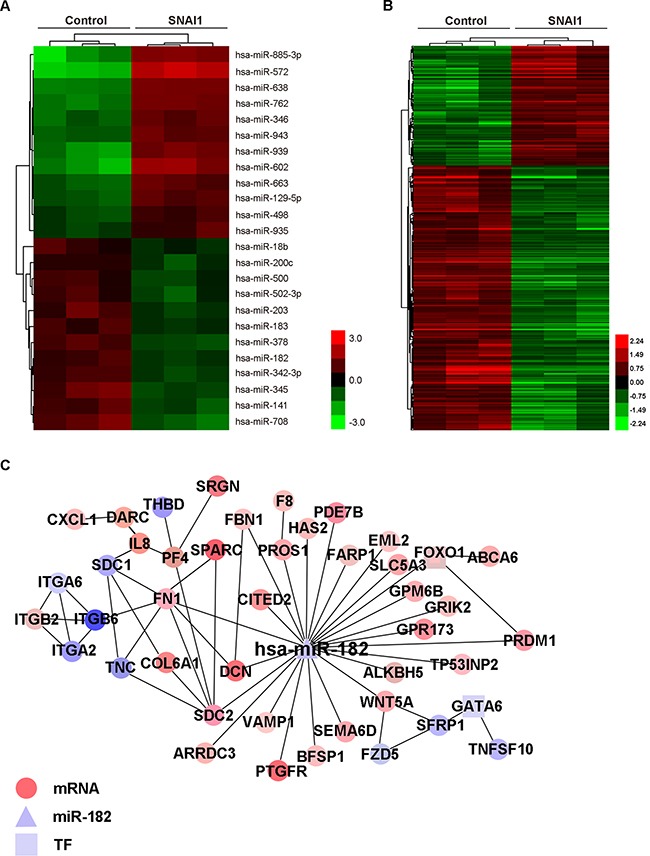
Identification of miR-182 in metastasis process **A-B**. Hierachical clustering of mainly dysregulated miRNAs (A) and gene microarray data (B) in MCF-10A cells with SNAI1 overexpression, in comparison with control. An expression ratio (log2) scale is shown. The red-green color-coded score indicates the significance level. The expression data were generated from three biological repetitions. **C**. miR-182/mRNA interaction network of MCF-10A with SNAI1 overexpression, generated by Cytoscape. miR-182 is depicted as a triangle, mRNAs as circles, and transcription factors (TFs) as squares. The effect of interaction (increase or decrease) is represented by colors, red for increase and blue for decrease.

To screen out more EMT-related miRNAs, we first induced an EMT model in MCF-10A cells with SNAI1 overexpression (Supplementary Figure S1). Then we comparatively analyzed miRNA microarray profiles in this EMT model. As shown in Figure 1A, microarray analysis revealed several mainly dsyregulated miRNAs in MCF-10A cells with SNAI1 overexpression. To further explore their roles in metastasis, we subsequently analyzed the variation of genes in the same model (Figure 1B) using cDNA microarray assays. A miRNA-mRNA regulatory network was predicted using bioinformatics methods (Supplementary Figure S2), based on the data of miRNA and mRNA microarray profiles. Some miRNAs and genes included in these profiles had been demonstrated to decrease or increase in EMT by previous reports, such as miR-200c, EpCAM and FN1 [27–29], or by confirmation of our group, such as miR-498 (Supplementary Figure S3A). Strikingly, miR-182 was predicted to directly inhibit some well-known genes, for example, FN1 (Supplementary data S3B) and integrins [29–30] (Figure 1C). Previous reports have demonstrated that miR-182 promotes metastasis in many different types of cancer, like colorectal, breast and liver cancer [31–33]. We are interested to know the correlation between the suppression of miR-182 in EMT and the promotion of metastasis.

### miR-182 is directly suppressed by SNAI1

**Figure 2:**
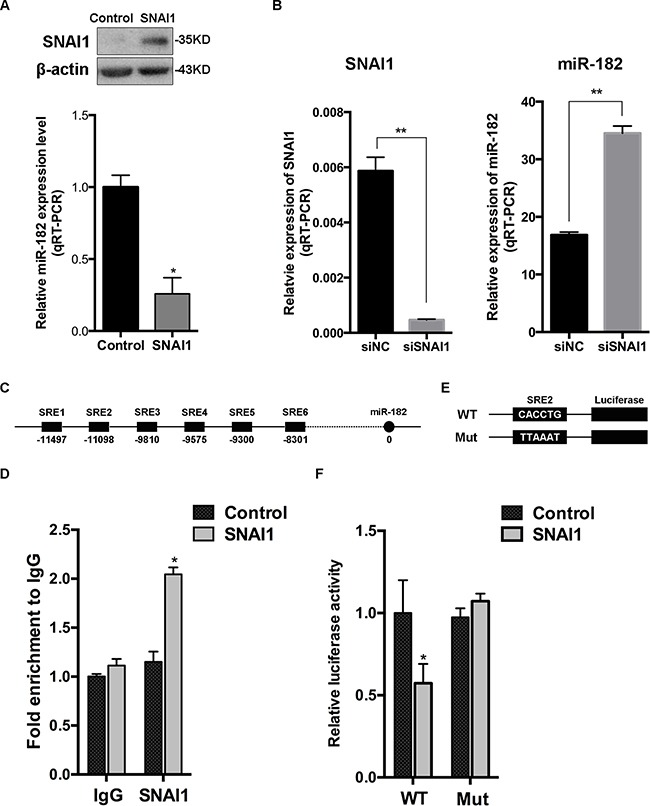
miR-182 is directly suppressed by SNAI1 **A**. SNAI1 protein level was detected in MCF-10A cells transfected with SNAI1 or a control vector, using Western Blot (up). Then, the endogenous expression of miR-182 in these two groups was examined using qRT-PCR (down). The values were normalized to U6. **B**. MCF-10A cells were transfected with SNAI1 siRNA (siSNAI1) or a control siRNA (siNC), then qRT-PCR was performed to detect the efficiency of SNAI1 knockdown (left) and the endogenous expression of miR-182 (right). The values were normalized to GAPDH and U6, respectively. **C**. Schematic of 6 typical SNAI1-responsive elements (SREs) of miR-182 promoter region using the CONSITE program. **D**. MCF-10A cells were transfected with SNAI1 or a control vector, then the ChIP assays were performed with antibody against SNAI1 or control IgG. The percentages of input of coprecipitating DNAs were detected by qPCR. Here shows the ChIP assay results for SRE2 of miR-182 promoter physically associated with SNAI1. **E**. A description of SRE2 of miR-182 promoter (WT) and its mutation (Mut) reporter constructs. **F**. Luciferase reporter assay of HEK293T cells co-transfected with SNAI1 and luciferase plasmids, that contained the SRE2 of miR-182 promoter in the form of either wild-type (WT) or mutation (Mut). Error bars in A, B, D and F represent mean ± SEM. *P<0.05. **P<0.01.

We first tested miR-182 expression in different breast cancer cell lines with SNAI1 overexpression, and found that miR-182 decreased in all these cell lines (Figure 2A and Supplementary Figure S4A). Contrarily, miR-182 increased in these cell lines with the diminishment of SNAI1 (Figure 2B and Supplementary Figure S4B), indicating that miR-182 was suppressed by SNAI1. As SNAI1 is a well-known transcriptional repressor [34], we wondered if SNAI1 would inhibit miR-182 in a direct manner. We analyzed miR-182 promoter region using the CONSITE program, and predicted six typical SNAI1-responsive elements (SREs, Figure 2C). The ChIP assay showed that SNAI1 could bind to SRE2, (Figure 2D, data of the rest SREs ChIP assay results are not shown), while mutated SRE2 led to the reduction (Figure 2E and 2F), indicating that SNAI1 represses miR-182 in a direct manner *via* binding to its promoter.

### miR-182 enables an epithelial-like state in breast cancer cells

**Figure 3:**
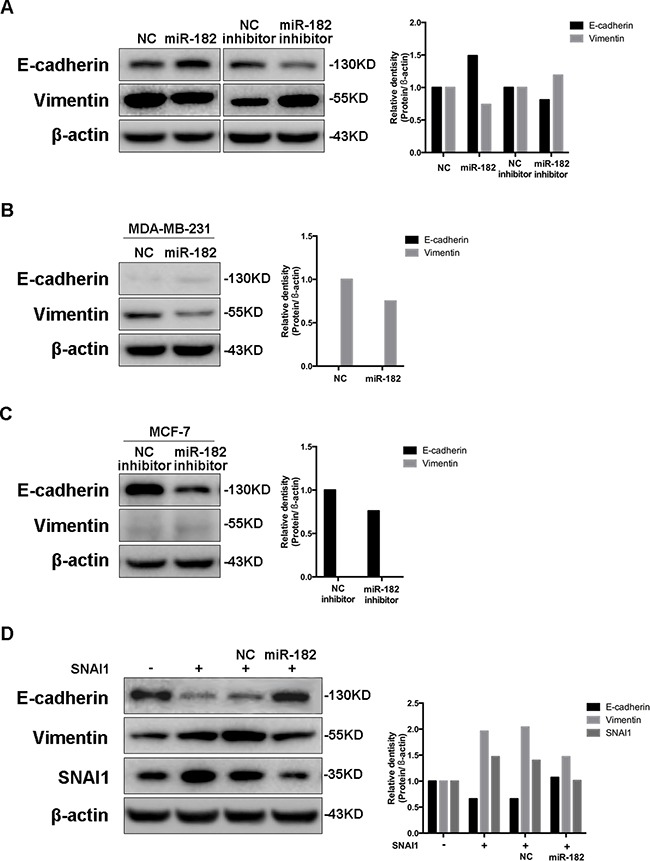
miR-182 enhances an epithelial-like state in breast cancer cells **A**. Western blot analysis of E-cadherin and Vimentin in MCF-10A cells transfected with either miR-182 mimics or inhibitor. **B-C**. The expression of E-cadherin and Vimentin in MDA-MB-231 cells transfected with miR-182 mimics (B) or in MCF-7 cells transfected with miR-182 inhibitor (C) was detected by Western blot. **D**. Western blot of E-cadherin, Vimentin and SNAI1 protein levels in MCF-10A cells transfected with a control or SNAI1 together with a NC mimics or miR-182 mimics. β-actin is shown as a loading control. The histogram on the right side represents the folder change of each gene expression detected by Western blot, compared with paralleled β-actin.

To examine the function of miR-182 as an EMT-related miRNA, we overexpressed miR-182 in MCF-10A cells, and observed an increase of E-cadherin and a decrease of Vimentin. Meanwhile, a contrary expression of these two proteins was detected with the diminishment of miR-182 (Figure 3A). Similar alterations were observed in 4T1 cells with the same treatment (Supplementary Figure S5). Even so, no morphological alteration was observed in either MCF-10A or 4T1 cells (Data not shown), suggesting that miR-182 could enable an epithelial-like state in these cells, rather than a complete EMT process. Moreover, overexpressing miR-182 in mesenchymal cancer cells decreased the expression of Vimentin (Figure 3B), while suppressing miR-182 in epithelial cancer cells reduced E-cadherin expression (Figure 3C). Furthermore, decrease of E-cadherin and increase of Vimentin in SNAI1-overexpressed MCF-10A could be reversed by miR-182 (Figure 3D). Likewise, no morphological alternation in all these cells was observed (Data not shown). These findings indicate that miR-182 enables an epithelial-like state in breast cancer cells when serving as an EMT-related miRNA.

### miR-182 promotes colonization and macroscopic metastasis formation

**Figure 4:**
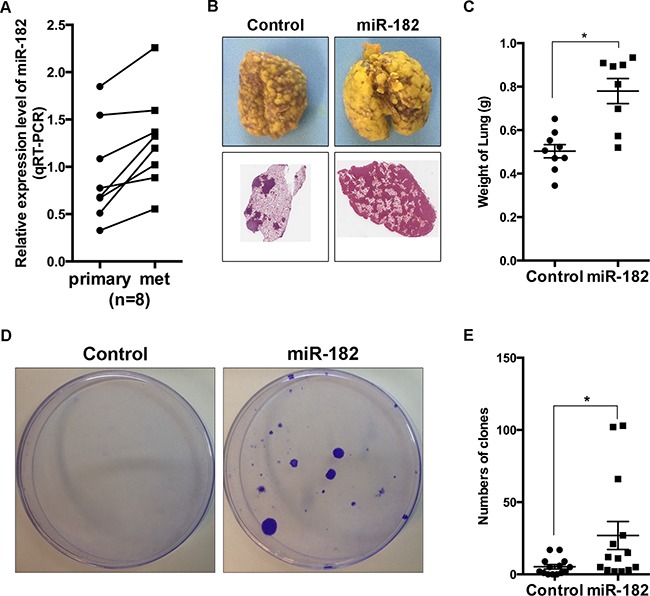
miR-182 promotes colonization and macrometastases formation at distant organs **A**. Parental 4T1 cells were orthotopically injected into mammary fat pads of female Balb/c-nu mice. Four weeks later, both primary tumors (primary) and macroscopic metastases in lungs (met) were excised. Expression of miR-182 in these specimens was detected, using qRT-PCR. The values were normalized to U6. **B**. 4T1 cells with a vector control or miR-182 overexpression were injected into female Balb/c-nu mice *via* tail vein, respectively. Three weeks later, the lungs were excised and fixed in bouins fixative. Representative gross lungs (upper) and H&E stained lung sections from these mice were shown. **C**. Quantitative analysis of metastasis by measuring the weight of the lungs burden metastasis. Mann-Whitney *U*-test was used to analyze the significance. **D**. 4T1 cells with a vector control or miR-182 overexpression were orthotopically injected into mammary fat pads, respectively. Colony formation assays were performed as described in Materials and Methods. Representative plate images of each group were shown. **E**. Numbers of colonies formed in colony formation assays. Error bars in C and E represent mean ± SEM. *P<0.05.

As mentioned previously, cancer cells need to reestablish their epithelial identity at distant site of metastasis [7], we wonder if the contribution of miR-182 on the epithelial-like state in breast cancer cells would have some effect on metastasis at distant site. Hence, we first inoculated 4T1 cells orthotopically into the mammary fat pads of Balb/c-nu mice, and examined miR-182 expression in both primary and spontaneous metastatic tumors. Compared with primary tumors, miR-182 elevated at metastatic site (Figure 4A). Simultaneously, the expression of E-cadherin was higher at metastatic site (Supplementary Figure S6), indicating that an epithelial identity is essential at metastatic site. Next, we overexpressed miR-182 in 4T1 cells, and introduced them directly into venous circulation for the measurement of pulmonary metastasis. As we expected, 4T1 cells overexpressing miR-182 resulted in enhanced metastasis potential to lung, compared with the control group (Figure 4B and 4C). Furthermore, miR-182-overexpressed 4T1 cells formed much more lung-derived tumor colonies when introduced orthotopically into mammary fat pads (Figure 4D and 4E, see Materials and Methods), suggesting that ectopic miR-182 expression can enhance the efficiency of lung colonization. Taken together, these results collectively show that miR-182 enhances colonization and macrometastases formation of breast cancer cells.

### SNAI1 is a direct and functional target of miR-182

**Figure 5:**
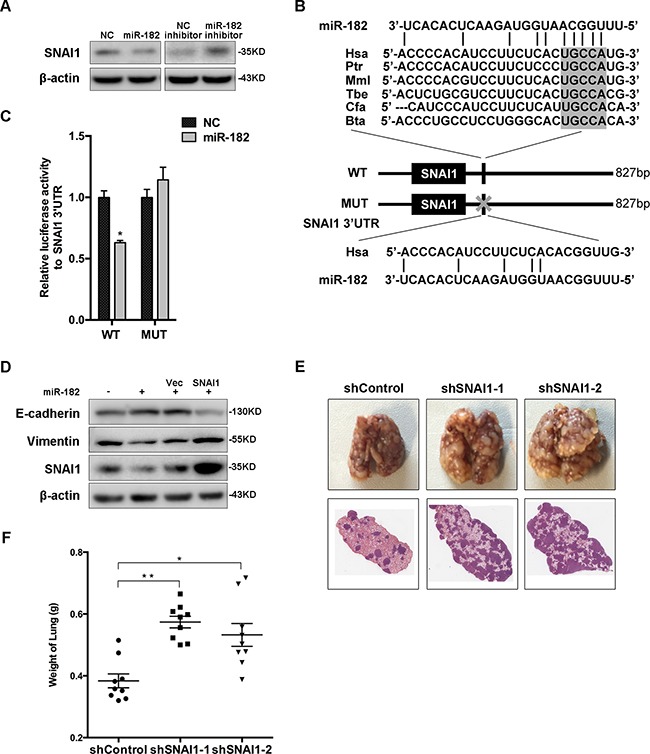
SNAI1 is a direct and functional target of miR-182 **A**. Western blot analysis of the protein levels of SNAI1 in MCF-10A cells transfected with miR-182 mimics or inhibitor. β-actin is shown as a loading control. **B**. A description of predicted miR-182 binding site within the SNAI1 3′UTR, as well as sequence alignment of the miR-182 binding site (WT) and its mutation (MUT) with the miR-182 targeting sequence. **C**. Luciferase reporter assay of HEK293T cells co-transfected with miR-182 mimics and either WT or Mut luciferase plasmid. **D**. ‘Rescue’ experiment was performed in MCF-10A cells transfected with NC or miR-182 mimics together with a control (Vec) or SNAI1 plasmid. The protein level of SNAI1 was analyzed using Western blot. β-actin is shown as a loading control. **E**. SNAI1 expression was blocked using two different targeting sequences in 4T1 cells, then injected into female Balb/c-nu mice via tail vein, respectively. Three weeks later, the lungs were excised and fixed in bouins fixative. Representative gross lungs (upper) and H&E stained lung sections from each group were shown. **F**. Measurement of weight of excised lungs, representing the macrometastases to lung. Mann-Whitney *U*-test was used to analyze the significance. Error bars in C and F represent mean ± SEM. *P<0.05, **P<0.01.

To identify novel functional targets of miR-182, we testified the predicted targets based on the microarray profiles (Figure 1C, Supplementary Figure S3C). Interestingly, a contrary alteration of SNAI1 expression was detected in both MCF-10A and 4T1 cells transfected with either miR-182 mimics or inhibitor (Figure 5A and Supplementary Figure S7). Analysis of SNAI1 3′UTR revealed a probable binding site complementary to miR-182 ‘seed sequence’, which was confirmed by luciferase reporter assay simultaneously (Figure 5B and 5C). Furthermore, the ‘Rescue’ experiment revealed a re-expression of SNAI1 in miR-182 overexpressed MCF-10A cells with the transfection of SNAI1, accompanied by the alternations of both E-cadherin and Vimentin expression (Figure 5D).

To further assess the functional role of SNAI1 as the target of miR-182, we established two SNAI1-knockdown subsets of 4T1. These subsets were then introduced into female Balb/c-nu mice via tail vein, respectively. Four weeks later, metastatic potential was assessed. As expected, diminishment of SNAI1 in these subsets enhanced metastasis burden in lung (Figure 5E and 5F). Together, the observations from both *in vitro* and *in vivo* analyses demonstrate that SNAI1 is a direct and functional target of miR-182.

### Clinical correlation of miR-182, SNAI1 and the epithelial state with metastatic colonization

**Figure 6:**
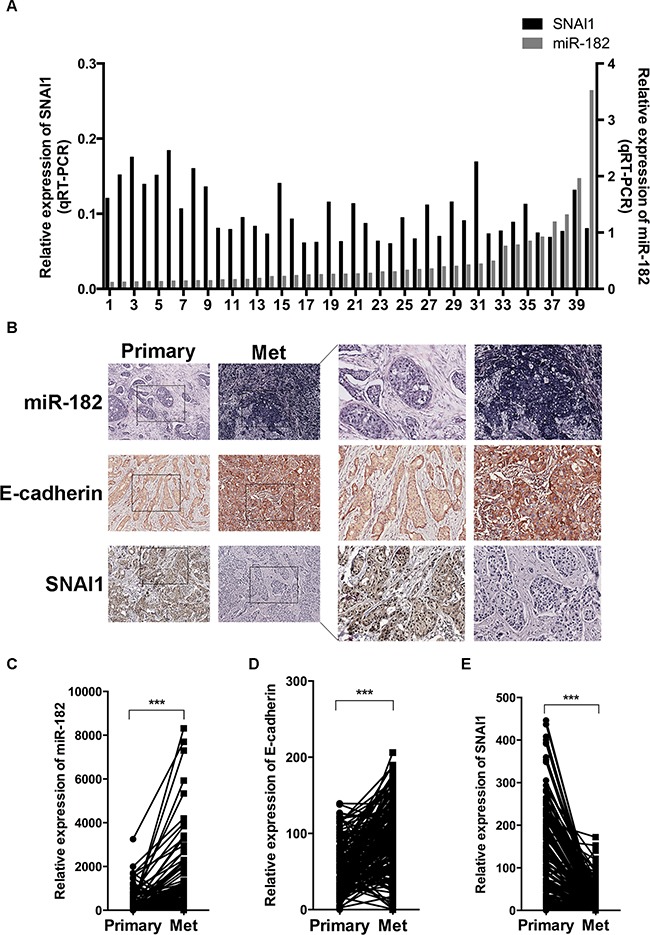
Correlation of miR-182 and SNAI1 expression with metastatic colonization **A**. Expression of miR-182 (gray, right Y axis) and SNAI1 (black, left Y axis) in 40 fresh frozen primary breast tumor specimens, examined by qRT-PCT. The values were normalized to U6 and GAPDH, respectively. **B-E**. Histological analysis of miR-182, E-cadherin and SNAI1 expression in primary breast cancer tissues (Primary) and paired metastatic lymph nodes (Met). The tissue microarray containing primary breast cancer tissues and paired metastatic lymph nodes was stained using a miR-182 probe (ISH) and specific antibody against E-cadherin or SNAI1 (IHC) as described in Materials and Methods, respectively. Here show representative photographs of ISH results of miR-182 and IHC results of E-cadherin and SNAI1 in breast cancer specimens (B), and the analysis of miR-182 (C), E-cadherin (D) and SNAI1 (E) expression in these specimens. Original magnification, ×400. *P<0.05, **P<0.01. ***P<0.001.

To further understand the clinical relevance of our findings to human breast cancer, we firstly examined both miR-182 and SNAI1 expression in 40 fresh frozen breast cancer tissue specimens, and found a significant inverse correlation between miR-182 and SNAI1 expression among these specimens (Figure 6A and Supplementary Figure S8), indicating a negative regulation between miR-182 and SNAI1 in tissues. Additionally, we collected 168 pairs of human breast cancer tissue specimens confirmed with positively metastatic lymph nodes, and examined both miR-182 and SNAI1 expression histologically (Figure 6B). Compared with primary tumors, a higher expression level of miR-182 was detected in paired metastatic lymph nodes (Figure 6B and 6C), whereas lower expression of SNAI1 was revealed in these metastatic lymph nodes (Figure 6B and Figure 6E). Besides, E-cadherin expression evaluated in metastatic tumor cells, implying an enhanced epithelial state in these cells (Figure 6B and 6D). These observations collectively point to correlation of miR-182, SNAI1 and the epithelial state in clinical metastasis, and the possibility that enabling an epithelial state by miR-182 may be crucial for macrometastases, with SNAI1 diminished conversely.

## DISCUSSION

EMT is always considered as the initial step of metastasis, nevertheless, metastatic cells need to reestablish the epithelial state for colonization and macrometastases formation at metastasis sites [3]. Previous studies have identified a large amount of miRNAs and genes involved in EMT [6, 35, 36]. However, the roles of these miRNAs and genes in colonization and macrometastases formation remain largely unknown. In this study, we identified miR-182 as an EMT-related miRNA based on miRNA microarray profiles. We found that miR-182 enhanced the epithelial-like state in breast cancer cells, as well as promoting significant colonization and macrometastasis formation in lungs when injecting directly into circulation.

miR-182 is a well-known oncomiR. A high level of miR-182 is correlated with metastasis and aggressiveness in a number of human cancers, such as melanomas, bladder cancer and liver cancer [33, 37–38]. In breast cancer, miR-182 is elevated and regulates metastasis positively [32, 39], suggesting that miR-182 might be an attractive therapeutic target in breast cancer. In our microarray profiles, miR-182 was characterized as an EMT inhibitor. Hence, we tried to find out the correlation between the decrease of miR-182 and the promotion of metastasis. We demonstrated that miR-182 was directly suppressed by SNAI1, a key transcription factor of EMT. However, we failed to observe any morphologic alteration in breast cancer cells with simply overexpressing or suppressing miR-182 expression, indicating that miR-182 could enable an epithelial-like state rather than reversing EMT process in breast cancer cells. Several researches have demonstrated that some EMT suppressors, such as miR-155 [21] and miR-200 [40], acted as promoters of colonization and macrometastases formation at distant organs, as well as reestablishing the epithelial identity in metastatic tumor cells. Given these, we wondered if miR-182 would play a similar role in breast cancer metastasis. As expected, we found that miR-182 promoted colonization and macrometastases formation at distant organs, via targeting SNAI1. It seems that reciprocal suppression between miR-182 and SNAI1 at different stages of metastasis forms a dynamic regulation loop, resulting in promoting metastasis.

Clinical observations revealed an increase of miR-182 and a decrease of SNAI1 in metastatic lymph nodes, accompanied by an enhanced epithelial identity. Both of the clinical and experimental findings suggest a reciprocal regulation between miR-182 and SNAI1 results in a promotion of metastasis. SNAI1 suppresses miR-182 at the stage of EMT to start metastasis, whereas miR-182 inhibits SNAI1 to reestablish epithelial identity for colonization and macrometastases formation. We observed the dynamic of miR-182/SNAI1 reciprocal suppression in metastasis process. However, the driven factors of this dynamic suppression need further exploration.

In summary, we demonstrated that miR-182 was directly repressed by SNAI1. It acted as an EMT suppressor, enabling an epithelial-like state in breast cancer cells. Moreover, miR-182 promoted colonization and macrometastases formation in lungs via inhibiting SNAI1. All our findings suggest a dynamic reciprocal suppression between miR-182 and SNAI1, resulting in promoting the complete process of metastasis.

## MATERIALS AND METHODS

### Cell culture

MCF-10A, MCF-7, MDA-MB-231 and HEK293T cells were obtained from the American Type Culture Collection (ATCC, Manassas, VA, USA) and maintained according to manufacturer's recommendations. 4T1 was maintained in RPMI 1640 supplemented with 10% fetal bovine serum and antibiotics.

### Plasmid construction

The 3′UTR of SNAI1 was cloned from MCF-10A complementary DNA, and the predicted promoter of miR-182 was cloned from MCF-10A genomic DNA. Then, these sequences were inserted into pIS0 and pGL3-basic luciferase plasmids, respectively. All the mutant sites were generated using a KOD-plus-Mutagenesis kit (Toyobo Co., LTD., Osaka, Japan). Pri-miR-182 amplified from MCF-10A genomic DNA and SNAI1 amplified from MCF-10A complementary DNA were inserted into pLVX-EGFP-C1, individually. For stable knockdown of SNAI1 (shSNAI1-1, shSNAI1-2), short hairpin RNA (shRNA) oligos containing the same targeting sequences as siRNA were synthesized, annealed and cloned into pSIH1-H1-Puro.

### Oligonucleotide and plasmid transfection

miR-182 mimics, inhibitor and relevant negative controls (NC for miR-182 mimics, NC inhibitor for miR-182 inhibitor) were synthesized by Invitrogen (Invitrogen, Camarillo, CA. USA). siRNA for SNAI1 and relevant negative control siNC were ordered from Qiagen (Qiagen, Germantown, MD, USA). Oligonucleotide and plasmid transfection were performed by using HiperFect (Qiagen) or Lipofectamine 2000 (Invitrogen) according to the manufacturer's recommendations. The sequences of oligonucleotides are shown in Supplementary Table S1.

### RNA isolation and quantitative PCR

Total RNA was extracted with TRIzol reagent (Invitrogen) and conventional quantitative RT-PCR was done using SYBR Premix Ex Taq (Takara, Dalian, China). Quantification of miR-182 was performed with a stem-loop real time PCR method as shown previously [13, 23]. snRNA U6 and glyceraldehyde-3-phophate dehydrogenase (GAPDH) were used as internal controls for microRNA and mRNA quantification, respectively. Primers for PCR are shown in Supplementary Table S2.

### Western blotting

Western blot analysis was performed as described previously [24, 25], and detected with the LAS4000 mini system (GE Healthcare, Piscataway, NJ, USA). Antibodies used to detect specific proteins were as following: β-actin (Sigma-Aldrich, St Louis, MO, USA, 1:4000), E-cadherin (Santa Cruz, Dallas, Texas, USA, 1:4000), Vimentin (Santa Cruz, 1:1000; Abnova, Taipei, Taiwan, 1:1000), SNAI1 (Abgent, San Diego, CA, USA, 1:1000; Abnova, 1:1000).

### Lentivirus production and transduction

Lentiviral vector and packaging plasmids were co-transfected into Lenti-293 cells using Lipofectamine 2000, respectively. The recombinant lentiviruses were harvested 48h after transfection. Breast cancer cells were infected with recombinant lentivirus-transducing units plus 5μg/ml Polybrene (Sigma-Aldrich). Stable clones were obtained using relevant agents for selection.

### Luciferase reporter assay

Luciferase reporter assay was done by using the Dual-Luciferase Reporter Assay System (Promega, Madison, Wisconsin, USA) as described previously [12, 26]. Each experiment was done in quadruplicate and repeated at least three times.

### Microarray assay

Three independent pairs of MCF-10A transduced with SNAI1 or control lentivirus were harvested with Trizol (Invitrogen). RNA samples were then subjected to microarray analysis using Affymetrix GeneChip miRNA 2.0 Array and Human Genome U133 Plus 2.0 Array (CapitalBio, Beijing, China). Raw data were processed by experts from CapitalBio before sending back to us. Raw data of the array experiment were submitted to Gene Expression Omnibus under GSE81931.

### Chromatin immunoprecipitation

Chromatin Immunoprecipitation (ChIP) analysis was performed using a Pierce® Agarose ChIP Kit (Thermo Fisher Scientific, Waltham, MA, USA). The antibody used was anti-SNAI1 from Santa Cruz. Primer sequences are shown in Supplementary Table S2

### *In situ* hybridization and immunohistochemistry

miR-182 LNA^TM^ probe was purchased from Exiqon (Vedbaek, Denmark), and *in situ* hybridization was performed as described previously [10, 12]. The paraffin-embedded breast cancer tissue array was stained with primary antibody against SNAI1 (Abnova, 1:50), and further colorized with a Universal Immuno-peroxidase Polymer Anti-Mouse/Rabbit Immunohistochemical Staining Reagent (ZSGB-BIO, Beijing, China), resulting in a brown signal. Images were visualized and annotated with Aperio ImageScope software (Aperio Technologies, Inc., CA, USA), and the number of positive cells at ×200 magnification was quantified.

### Clinical specimens

For fresh frozen breast cancer tissues, a total of 40 infiltrating ductal carcinoma samples were collected from November 2011 to November 2013 at Chinese Academy of Medical Sciences Cancer Hospital at the time of surgery and immediately stored in liquid nitrogen until use. For immunochemical analysis, 168 consecutive patients with histologically-confirmed breast cancer and paired-metastatic lymph node samples were all collected in 2006 at Harbin Medical University Cancer Hospital. These clinical samples were made into paraffin-embedded tissue array by Outdo Biotech (Outdo Biotech Co. Ltd., Shanghai, China). None of the patients had received chemotherapy or radiotherapy before surgery. Clinicopathological characteristics were shown in Table 1. The study was approved by both of the ethical committees of Chinese Academy of Medical Sciences Cancer Hospital and Harbin Medical University. Informed consent was obtained from all the patients.

**Table 1:** Clinicopathological characteristics of the patients

Clinicopathologic various	Number of cases
Patients at Chinese Academy of Medical Sciences Cancer Hospital	
Age	
<45 yr	10 (25.0%)
≥45 yr	30(75.0%)
ER	
Positive	25(62.5%)
Negative	15(37.5%)
PR	
Positive	24(60.0%)
Negative	16(40.0%)
Her2	
Positive	23(57.5%)
Negative	17(42.5%)
Lymph node metastasis	
Yes	22(55.0%)
No	18(45.0%)
Patients at Harbin Medical University Cancer Hospital	
Age	
<45 yr	52(31.0%)
≥45 yr	116(69.0%)
ER	
Positive	71(42.3%)
Negative	97(57.7%)
PR	
Positive	92(54.8%)
Negative	76(45.2%)
Her2	
Positive	117(69.6%)
Negative	51(30.4%)

ER, estrogen receptor; PR, progesterone receptor.

### Animal studies

All research involving animals complied with protocols approved by the Beijing Medical Experimental Animal Care Commission. For macrometastases, 1×10^5^ 4T1 cells infected with Control, miR-182 or shControl, shSNAI1-1, shSNAI1-2 were introduced into the circulation of six-week-old female Balb/c-nu mice via tail vein injection. Four weeks later, the lungs were excised, fixed in bouins fixative. The numbers of overt macrometastases were counted manually on fixed lungs, and then, these lungs were weighted, embedded in paraffin, sectioned and stained with hematoxylin and eosin (H&E). To quantify micrometastases, mice were sacrificed two weeks following orthotopic inoculation, lungs were excised, minced, digested and plated in puromycin selection in two 15cm tissue culture plates (serve as duplicates). Following two weeks of selection, tumor colonies were stained with crystal violet prior to counting.

### Statistical analyses

Statistical analyses were performed using Prism 6 (GraphPad Software, La Jolla, USA) or SPSS 20.0 (IBM SPSS software, NY, USA). All data are presented as mean ± SEM, unless otherwise stated. For animal assays, the Mann-Whitney *U*-test was used to determine the significance. The Student's *t*-test was used unless stated particularly (Spearman's correlation). All experiments other than histological and animal assays were repeated at least twice. We considered *p*< 0.05 as significant.

## SUPPLEMENTARY FIGURES AND TABLES


