# Supplementary material for: MicroRNA-182 drives colonization and macroscopic metastasis via targeting its suppressor SNAI1 in breast cancer
Source: Oncotarget. 2016 Nov 24;8(3):4629–41. doi: 10.18632/oncotarget.13542 (PMC5354860; doi:10.18632/oncotarget.13542)
Supplement: Supplementary file 1 [file oncotarget-08-4629-s001.pdf]

MicroRNA-182 drives colonization and macroscopic metastasis via targeting its suppressor SNAI1 in breast cancer

SUPPLEMENT\ARY TABLES AND FIGURES

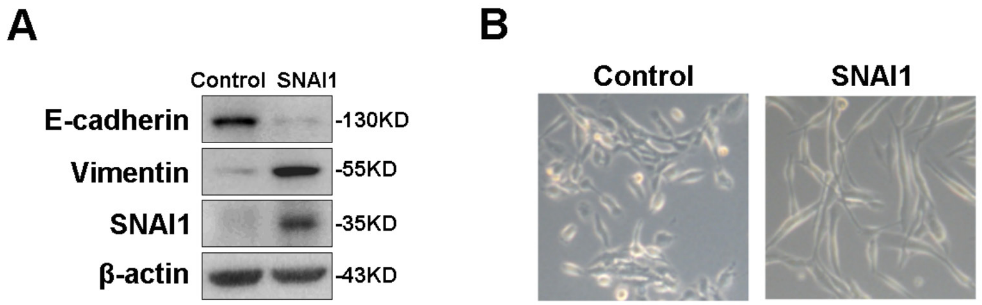

Supplementary Figure S1: EMT induced by overexpressing SNAI1. We overexpressed SNAI1 in MCF-10A cells. **A.** Western blot analysis was used to detect the expression of EMT-related markers, E-cadherin and Vimentin. β-actin is shown as a loading control. **B.** Images of cell morphology of MCF-10A cells with or without SNAI1 overexpression.

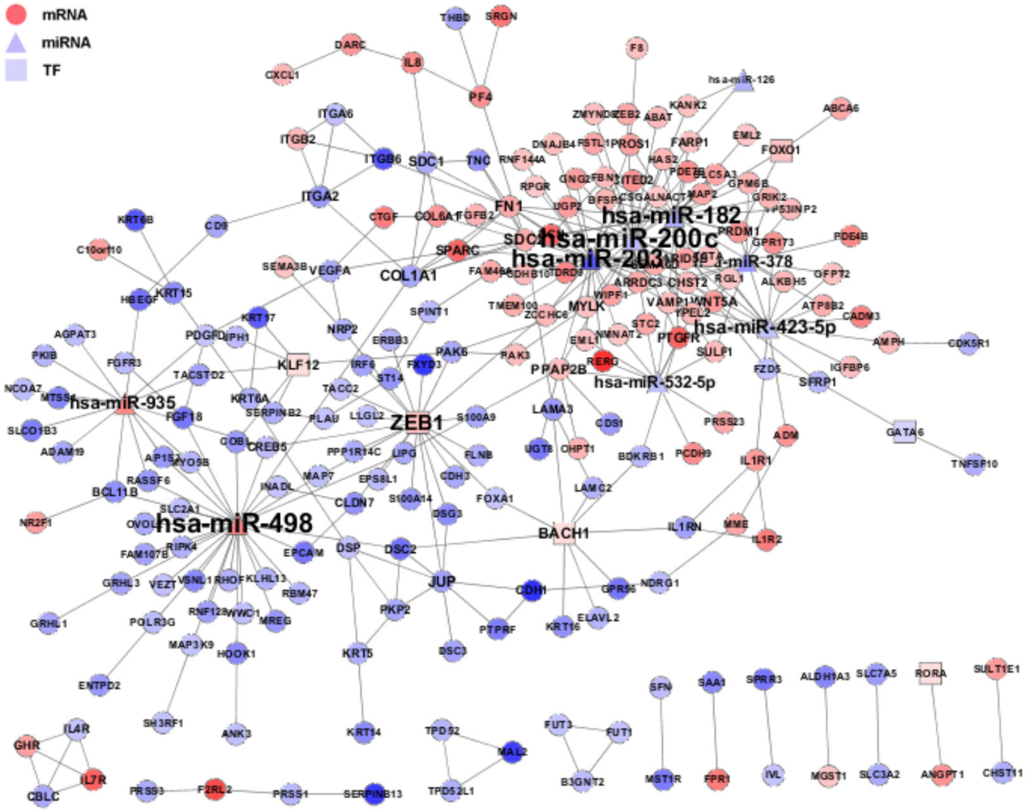

Supplementary Figure S2: ***In silico*** **analysis of miRNA and mRNA dysregulated in the process of SNAI1-induced EMT.** Integrative miRNA/mRNA network analysis of MCF-10A with SNAI1 overexpression, using Cytoscape. miRNAs are depicted as triangles, mRNAs as circles, and transcription factors (TFs) as squares. The effect of the interaction (upregulation or downregulation) is represented by spots in red (upregulation) and blue (downregulation).

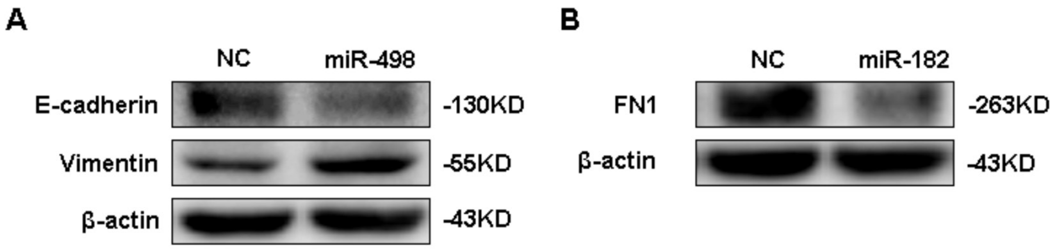

Supplementary Figure S3: Confirmation of the effectiveness of microarray profile data using positive controls. A. Microarray profiles showed an increase of miR-498 in SNAI1-induced EMT process. miR-498 mimics or NC was transfected into MCF-10A cells, then, the expression of E-cadherin and Vimentin were detected using Western blot analysis. **B.** FN1 was increased in SNAI1-induced EMT process, based on microarray profile data. Meanwhile, it was predicted to be suppressed by miR-182. Here we detected the protein level of FN1 in MCF-10A cells transfected with miR-182 mimics or NC, using Western blot analysis. β-actin is shown as a loading control in both A and B.

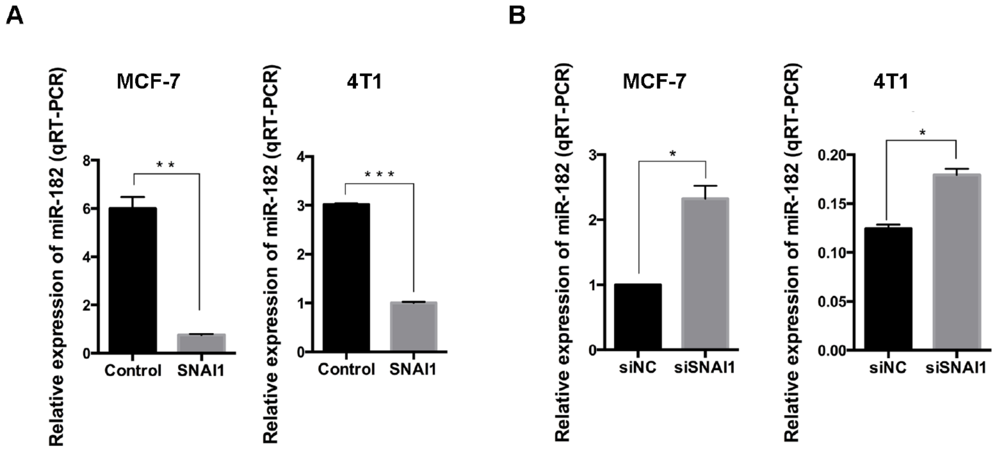

Supplementary Figure S4: miR-182 is directly suppressed by SNAI1. A. SNAI1 was overexpressed in MCF-7 and 4T1 cells, individually. Then, qRT-PCR was used to detect the endogenous expression of miR-182. The values were normalized to U6. **B.** Detection of endogenous miR-182 expression in MCF-7 and 4T1 cells transfected with SNAI1 siRNA or a control siRNA, using qRT-PCR. The values were normalized to U6. Error bars in this figure represent mean ± SEM. *$P<0.05$. **$P<0.01$. ***$P<0.001$.

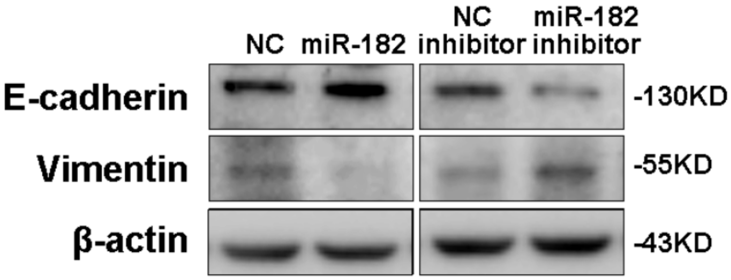

Supplementary Figure S5: miR-182 enhances an epithelial-like state in breast cancer cells. Western blot analysis of E-cadherin and Vimentin in 4T1 cells transfected with either miR-182 mimics or inhibitor. β-actin is shown as a loading control.

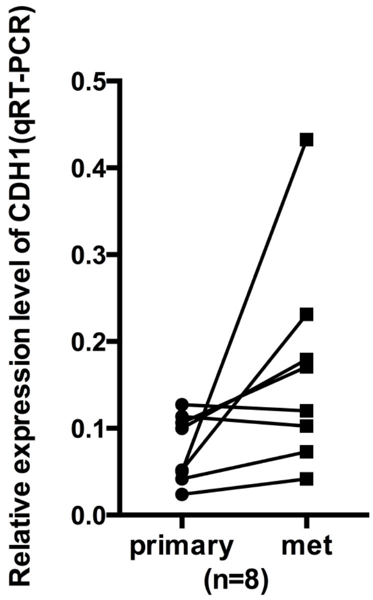

Supplementary Figure S6: E-cadherin increased in macrometastases. 4T1 cells were orthotopically injected into mammary fat pads of female Balb/c-nu mice. Four weeks later, both of the primary tumors (primary) and macrometastases in lungs (met) were excised. qRT-PCR was carried out to detect E-cadherin expression in these specimens. The values were normalized to GAPDH.

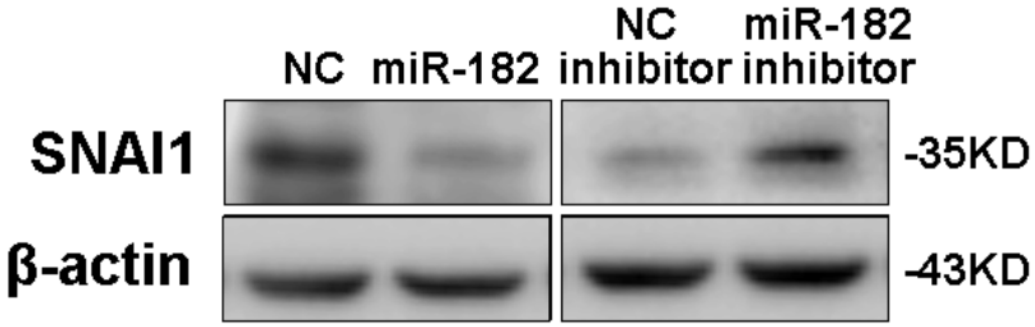

Supplementary Figure S7: SNAI1 is a direct and functional target of miR-182. Western blot analysis of the protein levels of SNAI1 in 4T1 cells transfected with miR-182 mimics or inhibitor. β-actin is shown as a loading control.

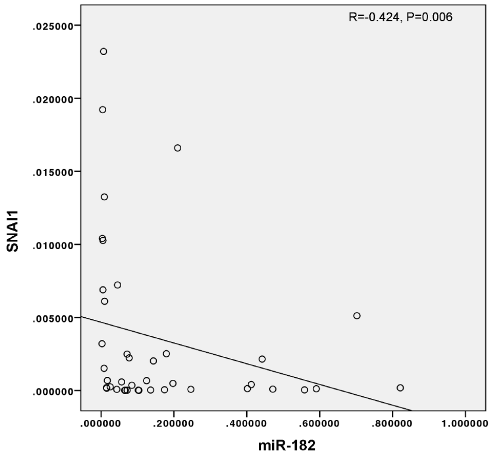

Supplementary Figure S8: Negative correlation between SNAI1 and miR-182 expression in breast cancer tissues. Inverse correlation between miR-182 and SNAI1 in 40 fresh frozen primary breast tumor specimens. A statistically significant correlation between miR-182 and SNAI1 expression was observed by Spearman's method with a correlation coefficient of -0.424 and *P* value of 0.006.

Supplementary Table S1: Oligonucleotides sequences used in this study.

Oligonucleotides	Sequences (from 5'-3')
NC	UUCUCCGAACGUGUCACGUTT<br>ACGUGACACGUUCGGAGAATT
miR-182 mimics	UUUGGCAAUGGUAGAACUCACACU<br>UGUGAGUUCUACCAUUGCCAAAUU
NC inhibitor	CAGUACUUUUGUGUAGUACAA
miR-182 inhibitor	AGUGUGAGUUCUACCAUUGCCAAA
siNC	AATTCTCCGAACGTGTCACGT
siSNAI1	GAGGTGTGACTAACTATGCAA<br>ACACTGGTATTTATATTTCAA<br>CCGAATGTCCCTGCTCCACAA<br>CAGCGAGCTGCAGGACTCTAA

Supplementary Table S2: Primers for PCR in this study.

Primers	Sequences (from 5'-3')
miR-182	F: ACACTCCAGCTGGGTTTGGCAATGGTAGAACT<br>R: TGGTGTCGTGGAGTCG
U6	F: CTCGCTTCGGCAGCACA<br>R: AACGCTTCACGAATTTGCGT
GAPDH	F: GGTGAAGGTCGGAGTCAACG<br>R: TGGGTGGAATCATATTGGAACA
SNAI1	F: CCTCCCTGTCAGATGAGGAC<br>R: CCAGGCTGAGGTATTCCTTG
mmu-GAPDH	F: TTGATGGCAACAATCTCCAC<br>R: CGTCCCGTAGACAAAATGGT
mmu-SNAI1	F: CTTGTGTCTGCACGACCTGT<br>R: AGTGGGAGCAGGAGAATGG
Primers for ChIP	
SRE1	F: AGATGGATCGGGAAGCAAGAR:<br>GCTCTGTCACGTGCTCATTA
SRE2	F: GGGCCTCCATGTCCTCACR:<br>GGGAGATGGGAACGGGTC
SRE3	F: CGGAGTGCACAGAGAATGACR:<br>TCAAGGTGAATCATCCCCGG
SRE4	F: GGGGAACAAAGGGGAGCCR:<br>CCCTGTTCTTCCGGTCCTC
SRE5	F: CCTCCGCCTCCTCTACTGAR:<br>TTTTGCCTGGCTTCTCTGGT
SRE6	F: TGACAGGTAGAGGGTGATGCR:<br>AAGCTCCTGAGGGCGAAAAT
